# 2-[(*E*)-2-(4-Eth­oxy­phen­yl)ethen­yl]-1-methyl­quinolinium 4-fluoro­benzene­sulfonate

**DOI:** 10.1107/S1600536813032509

**Published:** 2013-12-04

**Authors:** Hoong-Kun Fun, Thawanrat Kobkeatthawin, Pumsak Ruanwas, Ching Kheng Quah, Suchada Chantrapromma

**Affiliations:** a X-ray Crystallography Unit, School of Physics, Universiti Sains Malaysia, 11800 USM, Penang, Malaysia; b Department of Chemistry, Faculty of Science, Prince of Songkla University, Hat-Yai, Songkhla 90112, Thailand

## Abstract

In the structure of the title salt, C_20_H_20_NO^+^·C_6_H_4_FO_3_S^−^, the 4-(eth­oxy­phen­yl)ethenyl unit is disordered over two positions with a refined site-occupancy ratio of 0.610 (6):0.390 (6). The cation is nearly planar, the dihedral angle between the quinolinium and benzene rings being 6.7 (4) and 1.7 (7)° for the major and minor components, respectively. The eth­oxy group is essentially coplanar with the benzene ring [C—O—C—C_methy_ = 177.1 (8) and 177.8 (12)° for the major and minor components, respectively]. In the crystal, cations and anions are linked into chains along the *b*-axis direction by C—H⋯O_sulfon­yl_ weak inter­actions. These chains are further connected into sheets parallel to (001) by C—H⋯O_sulfon­yl_ weak inter­actions. The chains are also stacked along the *a* axis through π–π inter­actions involving the quinolinium and benzene rings [centroid–centroid distances = 3.636 (5) Å for the major component and 3.800 (9) Å for the minor component]. C—H⋯π inter­actions are also present.

## Related literature   

For background to the bioactivity and non-linear optical properties of quinolinium derivatives, see: Chanawanno *et al.* (2010 ▶); Hopkins *et al.* (2005 ▶); Musiol *et al.* (2006 ▶); O’Donnell *et al.* (2010 ▶); Ruanwas *et al.* (2010 ▶). For related structures, see: Chantrapromma *et al.* (2011 ▶); Fun *et al.* (2010 ▶); Ruanwas *et al.* (2010 ▶). For bond-length data, see: Allen *et al.* (1987 ▶). For the stability of the temperature controller used in the data collection, see: Cosier & Glazer (1986 ▶).
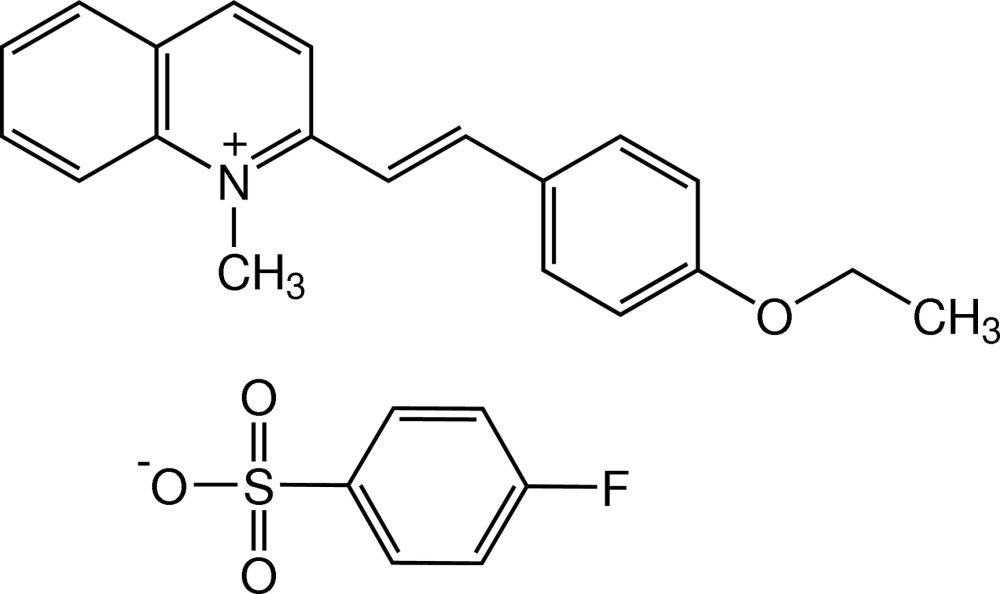


## Experimental   

### 

#### Crystal data  

C_20_H_20_NO^+^·C_6_H_4_FO_3_S^−^
*M* *_r_* = 465.52
Monoclinic, 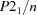

*a* = 6.4366 (3) Å
*b* = 9.8909 (5) Å
*c* = 34.3628 (15) Å
β = 95.102 (2)°
*V* = 2179.00 (18) Å^3^
*Z* = 4
Mo *K*α radiation
μ = 0.19 mm^−1^
*T* = 100 K
0.37 × 0.12 × 0.05 mm

#### Data collection  

Bruker APEXII CCD area-detector diffractometer
Absorption correction: multi-scan (*SADABS*; Bruker, 2009 ▶) *T*_min_ = 0.932, *T*_max_ = 0.991
19050 measured reflections
4993 independent reflections
3609 reflections with *I* > 2σ(*I*)
*R*_int_ = 0.060

#### Refinement  

*R*[*F* ^2^ > 2σ(*F* ^2^)] = 0.065
*wR*(*F* ^2^) = 0.154
*S* = 1.09
4993 reflections
392 parameters
418 restraints
H-atom parameters constrained
Δρ_max_ = 0.39 e Å^−3^
Δρ_min_ = −0.49 e Å^−3^

### 

Data collection: *APEX2* (Bruker, 2009 ▶); cell refinement: *SAINT* (Bruker, 2009 ▶); data reduction: *SAINT*; program(s) used to solve structure: *SHELXTL* (Sheldrick, 2008 ▶); program(s) used to refine structure: *SHELXTL*; molecular graphics: *SHELXTL*; software used to prepare material for publication: *SHELXTL*, *PLATON* (Spek, 2009 ▶), *Mercury* (Macrae *et al.*, 2006 ▶) and *publCIF* (Westrip, 2010 ▶).

## Supplementary Material

Crystal structure: contains datablock(s) global, I. DOI: 10.1107/S1600536813032509/rz5097sup1.cif

Structure factors: contains datablock(s) I. DOI: 10.1107/S1600536813032509/rz5097Isup2.hkl

Supporting information file. DOI: 10.1107/S1600536813032509/rz5097Isup3.cml

Additional supporting information:  crystallographic information; 3D view; checkCIF report

## Figures and Tables

**Table 1:** Hydrogen-bond geometry (Å, °) *Cg*4 and *Cg*5 are the centroids of the C12*B*–C17*B* and C21–C26 rings, respectively.

*D*—H⋯*A*	*D*—H	H⋯*A*	*D*⋯*A*	*D*—H⋯*A*
C2—H2*A*⋯O2^i^	0.93	2.55	3.456 (4)	166
C8—H8*A*⋯O4^ii^	0.93	2.41	3.306 (3)	161
C10—H10*A*⋯O3	0.96	2.55	3.483 (4)	164
C11*A*—H11*A*⋯O4^ii^	0.93	2.52	3.408 (19)	159
C17*A*—H17*A*⋯O3	0.93	2.58	3.510 (10)	177
C20—H20*B*⋯O2^i^	0.96	2.53	3.441 (4)	158
C20—H20*C*⋯O3	0.96	2.44	3.085 (4)	124
C25—H25*A*⋯O4^iii^	0.93	2.55	3.264 (4)	134
C13*A*—H13*A*⋯*Cg*5^ii^	0.93	2.82	3.575 (10)	139
C16*A*—H16*A*⋯*Cg*5	0.93	2.98	3.826 (9)	151
C19*A*—H19*B*⋯*Cg*4^iii^	0.96	2.99	3.862 (11)	152
C13*B*—H13*B*⋯*Cg*5^ii^	0.93	2.95	3.765 (16)	147
C16*B*—H16*B*⋯*Cg*5	0.93	2.70	3.562 (13)	155

Symmetry codes: (i) 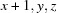; (ii) 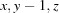; (iii) 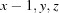.

**Fig. 1.**
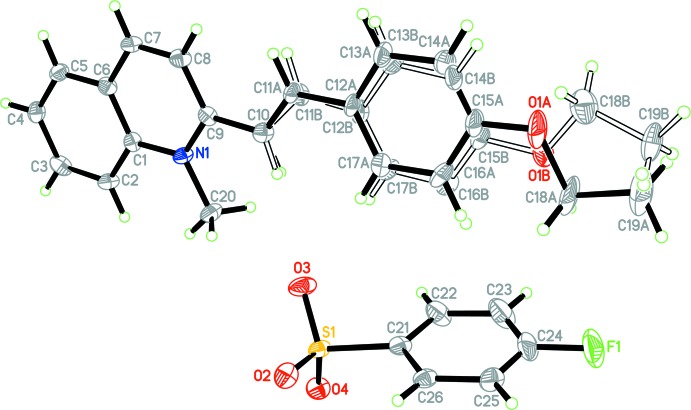
The structure of the title compound showing 50% probability displacement ellipsoids and the atom-numbering scheme. Open bonds show the minor component.

**Fig. 2.**
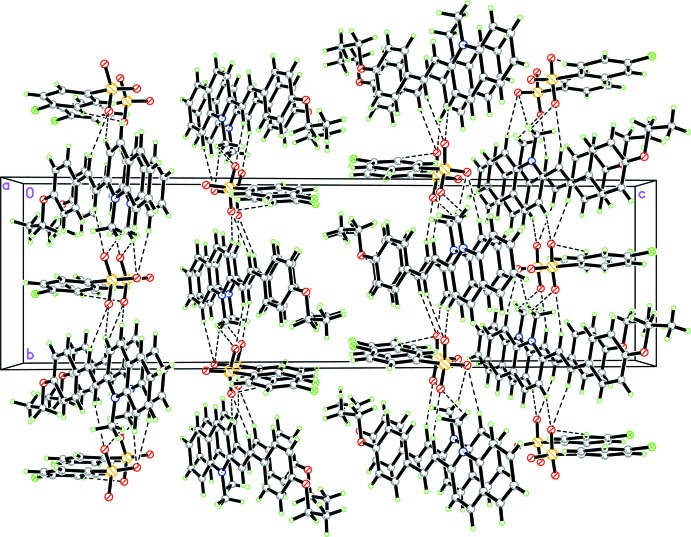
The crystal packing of the major component of the title compound viewed approximately along the a axis. Hydrogen bonds are drawn as dashed lines.

**Fig. 3.**
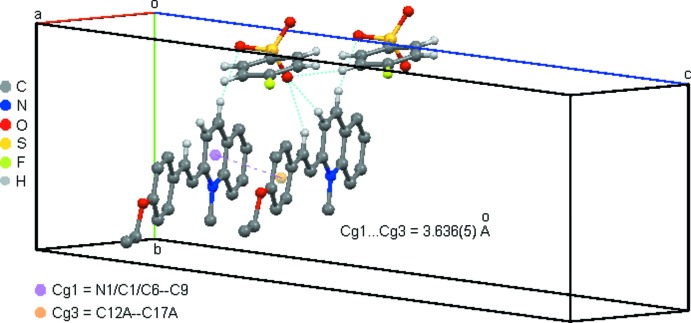
π–π interaction between aromatic rings of the cations of the major component. H-atoms of the cations not involved in hydrogen bonds are omitted for clarity.

## supplementary crystallographic information

### 1. Comment

Quinolinium derivatives were reported to possess interesting bioactivities and pharmacological activities (Chanawanno *et al.*, 2010; Hopkins *et al.*, 2005; Musiol *et al.*, 2006; O'Donnell *et al.*, 2010), including non-linear optic properties (Ruanwas *et al.*, 2010). During the course of our research on the antibacterial activity of pyridinium and quinolinium salts, the title quinolinium salt (I) was synthesized in order to study the effect of the anion counter-part on its antibacterial activity because its starting quinolinium iodide salt (Chanawanno *et al.*, 2010) was found to be very active against the methicillin-resistant *Staphylococcus aureus* with a MIC value of 2.34 µg/ml. Herein the synthesis and crystal structure of (I) are reported.

In the title salt (Fig. 1), C_20_H_20_NO^+^.C_6_H_4_FSO_3_^-^, the 4-(ethoxyphenyl)ethenyl unit is disordered over two positions with a refined site-occupancy ratio of 0.610 (6):0.390 (6). The cation exists in an *E* configuration with respect to the ethenyl bond [C10 ═C11 = 1.326 (18) Å for the major *A* component and 1.38 (3) Å for the minor *B* component] and torsion angle C9—C10—C11—C12 = -178.3 (12) ° for the major *A* component, and -179.0 (19)° for the minor *B* component. The 1-methylquinolinium ring system is planar with a *rms* deviation of 0.0199 (3) Å for the eleven non-H atoms. The cation is planar with dihedral angles between the N1/C1–C9 quinolinium and C12–C17 benzene rings of 6.7 (4) and 1.7 (7)° for the major *A* and minor *B* components, respectively. The ethoxy unit is disordered over two positions in such a way that the major *A* and minor *B* components are related by a 180° rotation. Moreover the ethoxy unit is co-planar with the attached benzene ring as indicated by the torsion angles C16–C15–O1–C18 = 2.5 (15)° and C15–O1–C18–C19 = 177.1 (8)° for the major *A* component. The corresponding values are 180.0 (14) and 177.8 (12)° for the minor *B* component. Bond distances in both cation and anion have normal values (Allen *et al.*, 1987) and are comparable to those observed in related structures (Chantrapromma *et al.*, 2011; Fun *et al.*, 2010; Ruanwas *et al.*, 2010).

In the crystal packing (Fig. 2), cations and anions are linked into chains along the *b* axis by C—H···O_sulfonyl_ weak interactions. These chains are further connected into sheets parallel to the (001) plane by C—H···O_sulfonyl_ weak interactions (Table 1), and these chains are also stacked by π–π interactions involving quinolinium and benzene rings (Fig. 3) with separations *Cg*_1_···*Cg*_3_^i^ = 3.636 (5) Å in the major component *A* and *Cg*_1_···*Cg*_4_^i^ = 3.800 (9) Å in the minor component *B* (symmetry code as in Table 1); *Cg*_1_, *Cg*_3_ and *Cg*_4_ are the centroids of the N1/C1/C6–C9, C12A–C17A and C12B–C17B rings, respectively. C—H···π interactions (Table 1) are also present.

### 2. Experimental

The title compound was synthesized by dissolving silver(I) 4-fluorobenzenesulfonate (0.20 g, 0.71 mmol) in methanol (20 ml) which upon heating was added to a solution of 2-[(*E*)-2-(4-ethoxyphenyl)ethenyl]-1-methylquinolinium iodide (Fun *et al.*, 2010) (0.29 g, 0.71 mmol) in hot methanol (30 ml). The mixture turned yellow and cloudy immediately. After stirring for 0.5 h, the precipitate of silver iodide which formed was filtered and the filtrate was evaporated to give a yellow solid. Yellow plate-shaped single crystals of the title compound suitable for X-ray structure determination were recrystallized from methanol by slow evaporation of the solvent at room temperature after a few weeks.

### 3. Refinement

All H atoms were positioned geometrically and allowed to ride on their parent atoms, with d(C-H) = 0.93 Å for aromatic and CH, 0.97 Å for CH_2_ and 0.96 Å for CH_3_ atoms. The *U*_iso_ values were constrained to be 1.5*U*_eq_ of the carrier atom for methyl H atoms and 1.2*U*_eq_ for the remaining H atoms. A rotating group model was used for the methyl groups. The 4-(ethoxyphenyl)-ethenyl unit is disordered over two sites with refined site occupancies ratio 0.610 (6):0.390 (6). Similarity and simulation restraints were applied.

### Crystal data

**Table:**

C_20_H_20_NO^+^·C_6_H_4_FO_3_S^−^	*F*(000) = 976
*M**_r_* = 465.52	*D*_x_ = 1.419 Mg m^−^^3^
Monoclinic, *P*2_1_/*n*	Mo *K*α radiation, λ = 0.71073 Å
Hall symbol: -P 2yn	Cell parameters from 4993 reflections
*a* = 6.4366 (3) Å	θ = 2.1–27.5°
*b* = 9.8909 (5) Å	µ = 0.19 mm^−^^1^
*c* = 34.3628 (15) Å	*T* = 100 K
β = 95.102 (2)°	Plate, yellow
*V* = 2179.00 (18) Å^3^	0.37 × 0.12 × 0.05 mm
*Z* = 4	

### Data collection

**Table:**

Bruker APEXII CCD area-detector diffractometer	4993 independent reflections
Radiation source: sealed tube	3609 reflections with *I* > 2σ(*I*)
Graphite monochromator	*R*_int_ = 0.060
φ and ω scans	θ_max_ = 27.5°, θ_min_ = 2.1°
Absorption correction: multi-scan (*SADABS*; Bruker, 2009)	*h* = −8→8
*T*_min_ = 0.932, *T*_max_ = 0.991	*k* = −12→11
19050 measured reflections	*l* = −44→44

### Refinement

**Table:**

Refinement on *F*^2^	Primary atom site location: structure-invariant direct methods
Least-squares matrix: full	Secondary atom site location: difference Fourier map
*R*[*F*^2^ > 2σ(*F*^2^)] = 0.065	Hydrogen site location: inferred from neighbouring sites
*wR*(*F*^2^) = 0.154	H-atom parameters constrained
*S* = 1.09	*w* = 1/[σ^2^(*F*_o_^2^) + (0.0388*P*)^2^ + 4.3613*P*] where *P* = (*F*_o_^2^ + 2*F*_c_^2^)/3
4993 reflections	(Δ/σ)_max_ < 0.001
392 parameters	Δρ_max_ = 0.39 e Å^−^^3^
418 restraints	Δρ_min_ = −0.49 e Å^−^^3^

### Special details

**Table:**

Experimental. The crystal was placed in the cold stream of an Oxford Cryosystems Cobra open-flow nitrogen cryostat (Cosier & Glazer, 1986) operating at 100.0 (1) K.
Geometry. All esds (except the esd in the dihedral angle between two l.s. planes) are estimated using the full covariance matrix. The cell esds are taken into account individually in the estimation of esds in distances, angles and torsion angles; correlations between esds in cell parameters are only used when they are defined by crystal symmetry. An approximate (isotropic) treatment of cell esds is used for estimating esds involving l.s. planes.
Refinement. Refinement of F^2^ against ALL reflections. The weighted R-factor wR and goodness of fit S are based on F^2^, conventional R-factors R are based on F, with F set to zero for negative F^2^. The threshold expression of F^2^ > 2sigma(F^2^) is used only for calculating R-factors(gt) etc. and is not relevant to the choice of reflections for refinement. R-factors based on F^2^ are statistically about twice as large as those based on F, and R- factors based on ALL data will be even larger.

### Fractional atomic coordinates and isotropic or equivalent isotropic displacement parameters (Å^2^)

**Table:**

	*x*	*y*	*z*	*U*_iso_*/*U*_eq_	Occ. (<1)
N1	1.5593 (3)	0.1235 (2)	0.18311 (7)	0.0173 (5)	
C1	1.7488 (4)	0.0857 (3)	0.20382 (8)	0.0170 (6)	
C2	1.8922 (4)	0.1828 (3)	0.22024 (9)	0.0211 (6)	
H2A	1.8655	0.2748	0.2173	0.025*	
C3	2.0733 (4)	0.1375 (3)	0.24085 (9)	0.0233 (6)	
H3A	2.1660	0.2008	0.2524	0.028*	
C4	2.1223 (4)	0.0006 (3)	0.24491 (9)	0.0214 (6)	
H4A	2.2460	−0.0267	0.2587	0.026*	
C5	1.9854 (4)	−0.0928 (3)	0.22830 (9)	0.0205 (6)	
H5A	2.0182	−0.1842	0.2303	0.025*	
C6	1.7957 (4)	−0.0528 (3)	0.20815 (8)	0.0184 (6)	
C7	1.6492 (4)	−0.1489 (3)	0.19255 (9)	0.0191 (6)	
H7A	1.6790	−0.2406	0.1952	0.023*	
C8	1.4663 (4)	−0.1090 (3)	0.17380 (9)	0.0198 (6)	
H8A	1.3698	−0.1738	0.1644	0.024*	
C9	1.4191 (4)	0.0300 (3)	0.16827 (8)	0.0163 (5)	
C10	1.2247 (4)	0.0745 (3)	0.14715 (9)	0.0197 (6)	
H10A	1.1959	0.1693	0.1442	0.024*	0.610 (6)
H10B	1.1971	0.1699	0.1466	0.024*	0.390 (6)
C20	1.5120 (5)	0.2681 (3)	0.17700 (10)	0.0245 (7)	
H20A	1.4912	0.2865	0.1495	0.037*	
H20B	1.6262	0.3213	0.1884	0.037*	
H20C	1.3876	0.2905	0.1891	0.037*	
O1A	0.3281 (9)	0.0972 (6)	0.04235 (19)	0.0386 (14)	0.610 (6)
C11A	1.086 (2)	−0.0139 (19)	0.1318 (6)	0.0169 (19)	0.610 (6)
H11A	1.1156	−0.1053	0.1356	0.020*	0.610 (6)
C12A	0.889 (2)	0.0214 (10)	0.1092 (4)	0.0174 (16)	0.610 (6)
C13A	0.7675 (19)	−0.0815 (10)	0.0915 (5)	0.0235 (16)	0.610 (6)
H13A	0.8105	−0.1708	0.0950	0.028*	0.610 (6)
C14A	0.5834 (16)	−0.0542 (8)	0.0687 (4)	0.0250 (16)	0.610 (6)
H14A	0.5068	−0.1244	0.0565	0.030*	0.610 (6)
C15A	0.5146 (15)	0.0770 (8)	0.0641 (4)	0.0246 (16)	0.610 (6)
C16A	0.6277 (15)	0.1820 (9)	0.0829 (3)	0.0246 (18)	0.610 (6)
H16A	0.5789	0.2703	0.0805	0.030*	0.610 (6)
C17A	0.8141 (15)	0.1543 (10)	0.1054 (3)	0.0190 (18)	0.610 (6)
H17A	0.8891	0.2246	0.1179	0.023*	0.610 (6)
C18A	0.2520 (9)	0.2376 (8)	0.03791 (18)	0.0459 (17)	0.610 (6)
H18A	0.3556	0.2933	0.0267	0.055*	0.610 (6)
H18B	0.2271	0.2745	0.0632	0.055*	0.610 (6)
C19A	0.0542 (11)	0.2371 (11)	0.0117 (2)	0.061 (2)	0.610 (6)
H19A	−0.0053	0.3262	0.0108	0.092*	0.610 (6)
H19B	−0.0423	0.1745	0.0216	0.092*	0.610 (6)
H19C	0.0832	0.2104	−0.0141	0.092*	0.610 (6)
O1B	0.3343 (14)	0.1530 (7)	0.0407 (3)	0.0238 (16)	0.390 (6)
C11B	1.076 (4)	−0.005 (3)	0.1270 (9)	0.019 (3)	0.390 (6)
H11B	1.1013	−0.0980	0.1272	0.023*	0.390 (6)
C12B	0.883 (3)	0.0408 (17)	0.1054 (8)	0.019 (2)	0.390 (6)
C13B	0.748 (3)	−0.0595 (16)	0.0880 (7)	0.022 (2)	0.390 (6)
H13B	0.7842	−0.1504	0.0903	0.026*	0.390 (6)
C14B	0.561 (3)	−0.0215 (13)	0.0672 (6)	0.022 (2)	0.390 (6)
H14B	0.4692	−0.0872	0.0564	0.027*	0.390 (6)
C15B	0.512 (2)	0.1117 (12)	0.0627 (6)	0.0188 (19)	0.390 (6)
C16B	0.647 (2)	0.2108 (13)	0.0783 (5)	0.020 (2)	0.390 (6)
H16B	0.6125	0.3016	0.0747	0.024*	0.390 (6)
C17B	0.831 (2)	0.1749 (15)	0.0993 (5)	0.017 (2)	0.390 (6)
H17B	0.9209	0.2421	0.1094	0.020*	0.390 (6)
C18B	0.1886 (13)	0.0568 (9)	0.0237 (3)	0.038 (2)	0.390 (6)
H18C	0.1379	−0.0008	0.0436	0.046*	0.390 (6)
H18D	0.2530	0.0005	0.0050	0.046*	0.390 (6)
C19B	0.0125 (13)	0.1380 (12)	0.0035 (3)	0.038 (2)	0.390 (6)
H19D	−0.0927	0.0778	−0.0080	0.057*	0.390 (6)
H19E	0.0647	0.1929	−0.0165	0.057*	0.390 (6)
H19F	−0.0468	0.1950	0.0223	0.057*	0.390 (6)
S1	0.97568 (10)	0.53446 (7)	0.16403 (2)	0.02062 (19)	
F1	0.3508 (3)	0.6116 (3)	0.03020 (7)	0.0605 (7)	
O2	0.8648 (3)	0.5242 (2)	0.19899 (6)	0.0262 (5)	
O3	1.0931 (3)	0.4139 (2)	0.15628 (7)	0.0337 (6)	
O4	1.0969 (3)	0.6584 (2)	0.16254 (6)	0.0261 (5)	
C21	0.7812 (4)	0.5484 (3)	0.12372 (9)	0.0223 (6)	
C22	0.8393 (5)	0.5361 (4)	0.08595 (10)	0.0331 (8)	
H22A	0.9763	0.5141	0.0820	0.040*	
C23	0.6945 (5)	0.5564 (4)	0.05408 (11)	0.0436 (10)	
H23A	0.7320	0.5488	0.0287	0.052*	
C24	0.4930 (5)	0.5883 (4)	0.06144 (11)	0.0378 (9)	
C25	0.4299 (5)	0.5984 (3)	0.09833 (10)	0.0286 (7)	
H25A	0.2922	0.6191	0.1021	0.034*	
C26	0.5755 (4)	0.5771 (3)	0.12989 (9)	0.0212 (6)	
H26A	0.5355	0.5819	0.1552	0.025*	

### Atomic displacement parameters (Å^2^)

**Table:**

	*U*^11^	*U*^22^	*U*^33^	*U*^12^	*U*^13^	*U*^23^
N1	0.0140 (11)	0.0130 (11)	0.0247 (13)	−0.0001 (8)	0.0009 (9)	−0.0002 (10)
C1	0.0109 (12)	0.0221 (13)	0.0181 (15)	0.0002 (10)	0.0016 (10)	−0.0002 (11)
C2	0.0206 (14)	0.0151 (13)	0.0278 (17)	0.0008 (11)	0.0031 (12)	−0.0024 (12)
C3	0.0174 (14)	0.0256 (15)	0.0268 (17)	−0.0101 (12)	0.0008 (12)	−0.0069 (13)
C4	0.0159 (13)	0.0268 (16)	0.0209 (16)	0.0014 (11)	−0.0011 (11)	0.0017 (12)
C5	0.0213 (14)	0.0170 (13)	0.0231 (16)	0.0033 (11)	0.0013 (12)	0.0011 (12)
C6	0.0154 (13)	0.0196 (14)	0.0201 (15)	−0.0023 (11)	0.0006 (11)	−0.0027 (12)
C7	0.0193 (14)	0.0132 (13)	0.0250 (16)	−0.0004 (10)	0.0031 (11)	−0.0014 (11)
C8	0.0167 (13)	0.0167 (13)	0.0261 (17)	−0.0056 (11)	0.0019 (12)	−0.0019 (12)
C9	0.0100 (12)	0.0209 (13)	0.0184 (14)	−0.0014 (11)	0.0026 (10)	−0.0023 (12)
C10	0.0144 (13)	0.0206 (14)	0.0242 (16)	0.0010 (11)	0.0020 (11)	−0.0012 (12)
C20	0.0198 (14)	0.0152 (13)	0.0371 (19)	0.0009 (11)	−0.0052 (13)	0.0016 (13)
O1A	0.019 (2)	0.065 (3)	0.030 (2)	0.010 (3)	−0.0085 (17)	0.005 (3)
C11A	0.013 (3)	0.019 (3)	0.018 (5)	0.005 (2)	−0.001 (3)	0.003 (3)
C12A	0.013 (2)	0.024 (3)	0.015 (3)	0.006 (2)	0.000 (2)	0.004 (3)
C13A	0.018 (3)	0.026 (3)	0.026 (3)	0.003 (2)	−0.006 (2)	0.005 (3)
C14A	0.019 (3)	0.027 (3)	0.029 (3)	0.004 (3)	−0.003 (2)	−0.002 (3)
C15A	0.016 (2)	0.035 (4)	0.022 (2)	0.006 (3)	−0.003 (2)	0.003 (3)
C16A	0.019 (3)	0.026 (4)	0.030 (3)	0.010 (3)	0.005 (2)	0.008 (3)
C17A	0.014 (3)	0.025 (4)	0.018 (3)	0.000 (2)	0.004 (2)	0.003 (3)
C18A	0.029 (3)	0.077 (4)	0.030 (3)	0.035 (3)	−0.002 (2)	0.009 (3)
C19A	0.037 (3)	0.110 (6)	0.036 (4)	0.033 (4)	−0.006 (3)	0.005 (4)
O1B	0.021 (3)	0.023 (3)	0.026 (3)	−0.008 (3)	−0.006 (2)	0.003 (3)
C11B	0.018 (4)	0.022 (5)	0.017 (5)	−0.004 (4)	0.000 (4)	0.002 (4)
C12B	0.011 (3)	0.027 (4)	0.019 (4)	0.000 (3)	0.001 (3)	0.003 (3)
C13B	0.021 (4)	0.018 (4)	0.025 (4)	0.003 (3)	−0.001 (3)	0.003 (4)
C14B	0.019 (4)	0.025 (4)	0.021 (3)	−0.002 (4)	−0.008 (3)	0.001 (4)
C15B	0.015 (3)	0.022 (4)	0.019 (3)	0.006 (3)	0.002 (3)	0.004 (3)
C16B	0.015 (3)	0.019 (4)	0.026 (4)	−0.001 (3)	0.005 (3)	0.005 (3)
C17B	0.013 (3)	0.015 (4)	0.023 (4)	−0.001 (3)	0.004 (3)	0.008 (3)
C18B	0.031 (4)	0.046 (4)	0.036 (4)	−0.007 (3)	−0.002 (3)	0.002 (4)
C19B	0.017 (4)	0.057 (5)	0.039 (5)	0.006 (4)	−0.007 (3)	0.013 (4)
S1	0.0126 (3)	0.0153 (3)	0.0329 (4)	0.0008 (3)	−0.0040 (3)	−0.0043 (3)
F1	0.0400 (13)	0.096 (2)	0.0406 (14)	0.0180 (13)	−0.0222 (10)	−0.0190 (13)
O2	0.0217 (10)	0.0269 (11)	0.0298 (13)	−0.0014 (9)	0.0015 (9)	0.0035 (10)
O3	0.0195 (11)	0.0232 (11)	0.0559 (16)	0.0070 (9)	−0.0105 (10)	−0.0129 (11)
O4	0.0193 (10)	0.0225 (11)	0.0355 (13)	−0.0056 (8)	−0.0031 (9)	−0.0040 (9)
C21	0.0145 (13)	0.0160 (13)	0.0353 (18)	−0.0031 (11)	−0.0050 (12)	−0.0068 (13)
C22	0.0163 (14)	0.049 (2)	0.0331 (19)	0.0027 (14)	−0.0005 (13)	−0.0184 (17)
C23	0.0313 (18)	0.067 (3)	0.032 (2)	0.0029 (18)	−0.0008 (15)	−0.0210 (19)
C24	0.0289 (17)	0.048 (2)	0.033 (2)	0.0046 (16)	−0.0146 (15)	−0.0118 (17)
C25	0.0139 (14)	0.0277 (16)	0.043 (2)	0.0015 (12)	−0.0054 (13)	−0.0066 (15)
C26	0.0171 (13)	0.0151 (13)	0.0311 (17)	−0.0014 (11)	0.0011 (12)	−0.0029 (12)

### Geometric parameters (Å, º)

**Table:**

N1—C9	1.359 (3)	C18A—H18A	0.9700
N1—C1	1.407 (3)	C18A—H18B	0.9700
N1—C20	1.473 (3)	C19A—H19A	0.9600
C1—C6	1.407 (4)	C19A—H19B	0.9600
C1—C2	1.414 (4)	C19A—H19C	0.9600
C2—C3	1.383 (4)	O1B—C15B	1.375 (11)
C2—H2A	0.9300	O1B—C18B	1.425 (10)
C3—C4	1.394 (4)	C11B—C12B	1.463 (12)
C3—H3A	0.9300	C11B—H11B	0.9300
C4—C5	1.366 (4)	C12B—C17B	1.380 (12)
C4—H4A	0.9300	C12B—C13B	1.417 (12)
C5—C6	1.406 (4)	C13B—C14B	1.399 (12)
C5—H5A	0.9300	C13B—H13B	0.9300
C6—C7	1.411 (4)	C14B—C15B	1.360 (11)
C7—C8	1.350 (4)	C14B—H14B	0.9300
C7—H7A	0.9300	C15B—C16B	1.385 (11)
C8—C9	1.418 (4)	C16B—C17B	1.377 (11)
C8—H8A	0.9300	C16B—H16B	0.9300
C9—C10	1.457 (4)	C17B—H17B	0.9300
C10—C11A	1.326 (18)	C18B—C19B	1.506 (10)
C10—C11B	1.38 (3)	C18B—H18C	0.9700
C10—H10A	0.9600	C18B—H18D	0.9700
C10—H10B	0.9600	C19B—H19D	0.9600
C20—H20A	0.9600	C19B—H19E	0.9600
C20—H20B	0.9600	C19B—H19F	0.9600
C20—H20C	0.9600	S1—O3	1.449 (2)
O1A—C15A	1.371 (7)	S1—O2	1.454 (2)
O1A—C18A	1.475 (8)	S1—O4	1.457 (2)
C11A—C12A	1.467 (8)	S1—C21	1.788 (3)
C11A—H11A	0.9300	F1—C24	1.367 (4)
C12A—C13A	1.392 (8)	C21—C22	1.388 (5)
C12A—C17A	1.403 (8)	C21—C26	1.389 (4)
C13A—C14A	1.388 (8)	C22—C23	1.388 (5)
C13A—H13A	0.9300	C22—H22A	0.9300
C14A—C15A	1.375 (8)	C23—C24	1.380 (5)
C14A—H14A	0.9300	C23—H23A	0.9300
C15A—C16A	1.394 (9)	C24—C25	1.369 (5)
C16A—C17A	1.394 (8)	C25—C26	1.385 (4)
C16A—H16A	0.9300	C25—H25A	0.9300
C17A—H17A	0.9300	C26—H26A	0.9300
C18A—C19A	1.492 (8)		
			
C9—N1—C1	121.7 (2)	C12A—C17A—H17A	119.7
C9—N1—C20	119.0 (2)	O1A—C18A—C19A	108.5 (6)
C1—N1—C20	119.3 (2)	O1A—C18A—H18A	110.0
C6—C1—N1	118.7 (2)	C19A—C18A—H18A	110.0
C6—C1—C2	119.5 (2)	O1A—C18A—H18B	110.0
N1—C1—C2	121.8 (2)	C19A—C18A—H18B	110.0
C3—C2—C1	118.3 (3)	H18A—C18A—H18B	108.4
C3—C2—H2A	120.8	C15B—O1B—C18B	120.8 (8)
C1—C2—H2A	120.8	C10—C11B—C12B	127 (2)
C2—C3—C4	122.6 (3)	C10—C11B—H11B	116.7
C2—C3—H3A	118.7	C12B—C11B—H11B	116.7
C4—C3—H3A	118.7	C17B—C12B—C13B	118.3 (11)
C5—C4—C3	118.9 (3)	C17B—C12B—C11B	124.3 (14)
C5—C4—H4A	120.6	C13B—C12B—C11B	117.2 (13)
C3—C4—H4A	120.6	C14B—C13B—C12B	119.8 (11)
C4—C5—C6	121.0 (3)	C14B—C13B—H13B	120.1
C4—C5—H5A	119.5	C12B—C13B—H13B	120.1
C6—C5—H5A	119.5	C15B—C14B—C13B	119.8 (11)
C5—C6—C1	119.6 (2)	C15B—C14B—H14B	120.1
C5—C6—C7	121.3 (3)	C13B—C14B—H14B	120.1
C1—C6—C7	119.1 (2)	C14B—C15B—O1B	121.5 (10)
C8—C7—C6	120.6 (3)	C14B—C15B—C16B	120.8 (10)
C8—C7—H7A	119.7	O1B—C15B—C16B	117.6 (9)
C6—C7—H7A	119.7	C17B—C16B—C15B	120.0 (10)
C7—C8—C9	121.0 (2)	C17B—C16B—H16B	120.0
C7—C8—H8A	119.5	C15B—C16B—H16B	120.0
C9—C8—H8A	119.5	C16B—C17B—C12B	121.1 (11)
N1—C9—C8	118.8 (2)	C16B—C17B—H17B	119.5
N1—C9—C10	119.6 (2)	C12B—C17B—H17B	119.5
C8—C9—C10	121.6 (2)	O1B—C18B—C19B	105.9 (8)
C11A—C10—C9	121.2 (6)	O1B—C18B—H18C	110.6
C11B—C10—C9	127.1 (10)	C19B—C18B—H18C	110.6
C11A—C10—H10A	119.1	O1B—C18B—H18D	110.6
C11B—C10—H10A	112.9	C19B—C18B—H18D	110.6
C9—C10—H10A	119.8	H18C—C18B—H18D	108.7
C11A—C10—H10B	121.6	C18B—C19B—H19D	109.5
C11B—C10—H10B	115.8	C18B—C19B—H19E	109.5
C9—C10—H10B	117.1	H19D—C19B—H19E	109.5
N1—C20—H20A	109.5	C18B—C19B—H19F	109.5
N1—C20—H20B	109.5	H19D—C19B—H19F	109.5
H20A—C20—H20B	109.5	H19E—C19B—H19F	109.5
N1—C20—H20C	109.5	O3—S1—O2	113.38 (14)
H20A—C20—H20C	109.5	O3—S1—O4	113.36 (13)
H20B—C20—H20C	109.5	O2—S1—O4	113.04 (13)
C15A—O1A—C18A	117.4 (6)	O3—S1—C21	105.18 (13)
C10—C11A—C12A	124.9 (12)	O2—S1—C21	106.51 (13)
C10—C11A—H11A	117.5	O4—S1—C21	104.35 (13)
C12A—C11A—H11A	117.5	C22—C21—C26	120.1 (3)
C13A—C12A—C17A	117.9 (7)	C22—C21—S1	119.3 (2)
C13A—C12A—C11A	118.9 (8)	C26—C21—S1	120.6 (2)
C17A—C12A—C11A	123.2 (9)	C21—C22—C23	120.5 (3)
C14A—C13A—C12A	121.6 (7)	C21—C22—H22A	119.8
C14A—C13A—H13A	119.2	C23—C22—H22A	119.8
C12A—C13A—H13A	119.2	C24—C23—C22	117.7 (3)
C15A—C14A—C13A	119.9 (7)	C24—C23—H23A	121.2
C15A—C14A—H14A	120.0	C22—C23—H23A	121.2
C13A—C14A—H14A	120.0	F1—C24—C25	118.8 (3)
O1A—C15A—C14A	117.2 (7)	F1—C24—C23	118.0 (3)
O1A—C15A—C16A	122.7 (7)	C25—C24—C23	123.2 (3)
C14A—C15A—C16A	120.0 (7)	C24—C25—C26	118.5 (3)
C15A—C16A—C17A	119.9 (7)	C24—C25—H25A	120.7
C15A—C16A—H16A	120.0	C26—C25—H25A	120.7
C17A—C16A—H16A	120.0	C25—C26—C21	120.0 (3)
C16A—C17A—C12A	120.5 (7)	C25—C26—H26A	120.0
C16A—C17A—H17A	119.7	C21—C26—H26A	120.0
			
C9—N1—C1—C6	1.9 (4)	O1A—C15A—C16A—C17A	178.5 (10)
C20—N1—C1—C6	−177.6 (3)	C14A—C15A—C16A—C17A	2.1 (16)
C9—N1—C1—C2	−178.2 (3)	C15A—C16A—C17A—C12A	0.1 (15)
C20—N1—C1—C2	2.4 (4)	C13A—C12A—C17A—C16A	−3.0 (17)
C6—C1—C2—C3	−1.3 (4)	C11A—C12A—C17A—C16A	178.8 (13)
N1—C1—C2—C3	178.7 (3)	C15A—O1A—C18A—C19A	177.1 (8)
C1—C2—C3—C4	2.1 (5)	C11A—C10—C11B—C12B	140 (19)
C2—C3—C4—C5	−0.7 (5)	C9—C10—C11B—C12B	−179.0 (19)
C3—C4—C5—C6	−1.5 (5)	C10—C11B—C12B—C17B	6 (4)
C4—C5—C6—C1	2.2 (4)	C10—C11B—C12B—C13B	−178 (3)
C4—C5—C6—C7	−176.9 (3)	C17B—C12B—C13B—C14B	−4 (3)
N1—C1—C6—C5	179.2 (3)	C11B—C12B—C13B—C14B	180 (2)
C2—C1—C6—C5	−0.8 (4)	C12B—C13B—C14B—C15B	2 (3)
N1—C1—C6—C7	−1.7 (4)	C13B—C14B—C15B—O1B	176.6 (19)
C2—C1—C6—C7	178.4 (3)	C13B—C14B—C15B—C16B	0 (3)
C5—C6—C7—C8	179.0 (3)	C18B—O1B—C15B—C14B	3 (2)
C1—C6—C7—C8	−0.2 (4)	C18B—O1B—C15B—C16B	180.0 (14)
C6—C7—C8—C9	1.9 (5)	C14B—C15B—C16B—C17B	−1 (3)
C1—N1—C9—C8	−0.2 (4)	O1B—C15B—C16B—C17B	−177.5 (16)
C20—N1—C9—C8	179.3 (3)	C15B—C16B—C17B—C12B	−1 (3)
C1—N1—C9—C10	179.8 (3)	C13B—C12B—C17B—C16B	3 (3)
C20—N1—C9—C10	−0.7 (4)	C11B—C12B—C17B—C16B	180 (2)
C7—C8—C9—N1	−1.7 (4)	C15B—O1B—C18B—C19B	177.8 (12)
C7—C8—C9—C10	178.3 (3)	O3—S1—C21—C22	−48.2 (3)
N1—C9—C10—C11A	178.9 (12)	O2—S1—C21—C22	−168.8 (2)
C8—C9—C10—C11A	−1.0 (12)	O4—S1—C21—C22	71.3 (3)
N1—C9—C10—C11B	173 (2)	O3—S1—C21—C26	134.5 (2)
C8—C9—C10—C11B	−7 (2)	O2—S1—C21—C26	13.9 (3)
C11B—C10—C11A—C12A	−36 (15)	O4—S1—C21—C26	−105.9 (2)
C9—C10—C11A—C12A	−178.3 (12)	C26—C21—C22—C23	2.0 (5)
C10—C11A—C12A—C13A	173.8 (18)	S1—C21—C22—C23	−175.2 (3)
C10—C11A—C12A—C17A	−8 (2)	C21—C22—C23—C24	−0.3 (6)
C17A—C12A—C13A—C14A	4 (2)	C22—C23—C24—F1	178.5 (3)
C11A—C12A—C13A—C14A	−177.8 (15)	C22—C23—C24—C25	−1.1 (6)
C12A—C13A—C14A—C15A	−2 (2)	F1—C24—C25—C26	−178.9 (3)
C18A—O1A—C15A—C14A	179.0 (10)	C23—C24—C25—C26	0.7 (6)
C18A—O1A—C15A—C16A	2.5 (15)	C24—C25—C26—C21	1.1 (4)
C13A—C14A—C15A—O1A	−177.8 (12)	C22—C21—C26—C25	−2.4 (4)
C13A—C14A—C15A—C16A	−1.2 (18)	S1—C21—C26—C25	174.8 (2)

### Hydrogen-bond geometry (Å, º)

*Cg*4 and *Cg*5 are the centroids of the C12B–C17B and C21–C26 rings, respectively.

**Table:**

*D*—H···*A*	*D*—H	H···*A*	*D*···*A*	*D*—H···*A*
C2—H2*A*···O2^i^	0.93	2.55	3.456 (4)	166
C8—H8*A*···O4^ii^	0.93	2.41	3.306 (3)	161
C10—H10*A*···O3	0.96	2.55	3.483 (4)	164
C11*A*—H11*A*···O4^ii^	0.93	2.52	3.408 (19)	159
C17*A*—H17*A*···O3	0.93	2.58	3.510 (10)	177
C20—H20*B*···O2^i^	0.96	2.53	3.441 (4)	158
C20—H20*C*···O3	0.96	2.44	3.085 (4)	124
C25—H25*A*···O4^iii^	0.93	2.55	3.264 (4)	134
C13*A*—H13*A*···*Cg*5^ii^	0.93	2.82	3.575 (10)	139
C16*A*—H16*A*···*Cg*5	0.93	2.98	3.826 (9)	151
C19*A*—H19*B*···*Cg*4^iii^	0.96	2.99	3.862 (11)	152
C13*B*—H13*B*···*Cg*5^ii^	0.93	2.95	3.765 (16)	147
C16*B*—H16*B*···*Cg*5	0.93	2.70	3.562 (13)	155

Symmetry codes: (i) *x*+1, *y*, *z*; (ii) *x*, *y*−1, *z*; (iii) *x*−1, *y*, *z*.
